# Identification of Input Nonlinear Control Autoregressive Systems Using Fractional Signal Processing Approach

**DOI:** 10.1155/2013/467276

**Published:** 2013-06-17

**Authors:** Naveed Ishtiaq Chaudhary, Muhammad Asif Zahoor Raja, Junaid Ali Khan, Muhammad Saeed Aslam

**Affiliations:** ^1^Department of Electronic Engineering, International Islamic University, Islamabad 44000, Pakistan; ^2^Department of Electrical Engineering, COMSATS Institute of Information Technology, Attock Campus, Attock 43600, Pakistan; ^3^Pakistan Institute of Engineering and Applied Sciences, Nilore, Islamabad 45650, Pakistan

## Abstract

A novel algorithm is developed based on fractional signal processing approach for parameter estimation of input nonlinear control autoregressive (INCAR) models. The design scheme consists of parameterization of INCAR systems to obtain linear-in-parameter models and to use fractional least mean square algorithm (FLMS) for adaptation of unknown parameter vectors. The performance analyses of the proposed scheme are carried out with third-order Volterra least mean square (VLMS) and kernel least mean square (KLMS) algorithms based on convergence to the true values of INCAR systems. It is found that the proposed FLMS algorithm provides most accurate and convergent results than those of VLMS and KLMS under different scenarios and by taking the low-to-high signal-to-noise ratio.

## 1. Introduction

Parameter estimation methods have been applied in many important applications arising in applied science and engineering including linear and nonlinear system identification, signal processing, and adaptive control [1–9]. Nonlinear systems are generally categorized into input, output, feedback, and hybrid, that is, combination of input and output nonlinear systems. Many nonlinear systems are modeled with Hammerstein model, a class of input nonlinear systems that consists of static nonlinear blocks followed by linear dynamical subsystems [10, 11]. Such models have been broadly used in diverse fields such as nonlinear filtering [12], biological systems [13], actuator saturations [14], chemical processes [15], audiovisual processing [16], and signal analysis [17]. 

A lot of interest has been shown by the research community for parameter estimation of Hammerstein nonlinear controlled autoregression models also known as input nonlinear controlled auto-regression (INCAR) systems. For instance, Ding and Chen have developed a least square based iterative procedure and an adaptive extended version of the least square algorithm for Hammerstein autoregressive moving average with exogenous inputs (ARMAX) system [18], Ding et al. also present an auxiliary model using recursive least square algorithm for Hammerstein output error systems [19], and Fan et al. have developed the least square identification algorithm for Hammerstein nonlinear autoregressive with exogenous inputs (ARX) models, while Wang and Ding have developed the extended stochastic gradient algorithm for Hammerstein-Wiener ARMAX models. As per authors' literature survey adaptive or recursive algorithms based on fractional signal processing approach like fractional least mean square algorithm (FLMS) and its normalized version have not been exploited in this domain. 

The application of fractional signal processing has been arising in many fields of science and technology including modeling of fractional Brownian motion [20], description of fractional damping [21], charge estimation of lead acid battery through identification of fractional systems [22], which differintegration [23], and Identifying a transfer function from a frequency response[24] etc. Fundamental description, subject terms, importance, and history of fractional signal process can be seen in [25, 26]. Wealth of information about fractional signal processing is also available in special issues of renewed journals [27, 28]. Fractional time integral approach to image structure denoising [29] and design for the adjustable fractional order differentiator [30] are other illustrative recent applications of these approaches. These are also motivation factors for the authors to explore applications of fractional signal processing specially in the area of Hammerstein nonlinear systems.

In this paper, adaptive algorithm based on fractional least mean square (FLMS) approach is applied for parameter estimation of INCAR model to find unknown parameter vector. The FLMS algorithm with different step size parameters is applied to two examples of INCAR model, and performance of the proposed scheme is analyzed for different scenarios of signal-to-noise ratios. The optimization problem is also adaptive with Volterra LMS and recently proposed kernel LMS, and comparison of the results is made with FLMS algorithm for each case of both examples. 

The organization of the paper is as follows; in Section 2 the description of the problem based on INCAR model is presented. In Section 3, proposed adaptive algorithms are described. Results of detailed simulations are given in Section 4 alone with necessary discussion. We conclude our finding in the last sections along with few future research directions in this domain. 

## 2. Input Nonlinear Control Autoregressive Systems

In this section, the brief description of input nonlinear control autoregressive (INCAR) systems is presented. 

Let us consider the following governing equation of INCAR model as [18, 31]

(1) P(z)y(t)=Q(z)u−(t)+v(t),

here *y*(*t*) represents the output of system, *v*(*t*) is the disturbance noise, $\overset{-}{u}\left(t\right)$ is output of nonlinear block and is given as a nonlinear function of *m* known basis (*f*_1_, *f*_2_, …, *f*_*m*_) of the system input *u*(*t*) as

(2) u−(t)=f(x(t))=a1f1(u(t))+a2f2(u(t))+⋯+amfm((t)),

where **A** = [*a*_1_,*a*_2_,…,*a*_*m*_]^*T*^ ∈ ℝ^*m*^ is the vector of constants, *P*(*z*) and *Q*(*z*) are known polynomials and given in term of unit backward shift operator *z* ^−1^[*z* ^−1^ *y*(*t*) = *y*(*t* − 1)], as

(3) P(z)=1+p1z−1+p2z−2+⋯+pnz−n,Q(z)=q1z−1+q2z−2+q3z−3+⋯+qnz−n,

where **p** = [*p*_1_,*p*_2_,…,*p*_*n*_]^*T*^ ∈ ℝ^*n*^ and **q** = [*q*_1_,*q*_2_,…,*q*_*n*_]^*T*^ ∈ ℝ^*n*^ are the constants coefficient vectors. Rearranging equation (1) one has

(4) y(t)=[1−P(z)]y(t)+Q(z)u−(t)+v(t)

Using (3) in (4) one has

(5) y(t)=−∑i=1npiy(t−i)+∑i=1n∑j=1mqiajfju(t−i)+v(t)=−∑i=1n(piy(t−i))+q1a1f1(u(t−1))  +q1a2f2(u(t−1))+⋯+q1amfm(u(t−1))  +q2a1f1(u(t−2))+q2a2f2(u(t−2))+⋯  +q2amfm(u(t−2))+⋯+qna1f1(u(t−n))  +qna2f2(u(t−n))+⋯  +qnamfm(u(t−n))+v(t)=φT(t)θ+v(t),

where the parameter vector ***θ*** and information vector *φ*(*t*) are defined as

(6) θ=[pT,q1aT,q2aT,…,qmaT]T∈ℝn0, n0=n+mn,φ(t)=[φ0T(t),φ1T(t),φ2T(t),…,φmT(t)]T∈ℝn0,                     n0=n+mn,φ0(t)=[−y(t−1),−y(t−2),…,y(t−n)]T∈ℝn,φj(t) =[fj(u(t−1)),fj(u(t−2)),…,fj(u(t−n))]T∈ℝn,                          j=1,2,…,m.

Equation (5) represents the linear-in-parameters identification model for Hammerstein control autoregressive systems using parameterization. The detail studies of input nonlinear systems, interested reader are referred to [32]. 

## 3. Methodologies for Parameter Estimation of INCAR Model 

In this section, brief introductory material is presented for proposed adaptive algorithms for identification of INCAR model given in Section 5. Three recursive algorithms are used: optimization of the model including fractional least mean square (FLMS), Volterra least mean square (VLMS), and kernel least mean square (KLMS).

### 3.1. Fractional Least Mean Square (FLMS) Algorithm

FLMS belongs to the class of nonlinear adaptive algorithms which is introduced by Zahoor and Qureshi [33] in their work of identification of autoregressive (AR) systems. Since origination of FLMS algorithm, it has been utilized immensely in various problems effectively such as dual-channel speech enhancement [34, 35], acoustic echo cancellation [36], and performance analysis of Bessel beamformers [37, 38]. Our intention is thin study to use FLMS with a different order for parameter estimation of INCAR systems.

The cost function for adaptive algorithm like FLMS is given as

(7) j(n)=E[|e(n)|2],

where

(8) e(n)=d(n)−y(n)

*e*(*n*) represents the difference between desired *d*(*n*) and *y*(*n*) filter response, *u*(*n*) is the input to the filter, and *μ* is the step size parameter.

Normally, the filter weight update equation for least mean square (LMS) algorithm is written as

(9) wk(n+1)=wk(n)−μ∂j(n)∂wk, k=0,1,2,…,M−1,

where *M* is the number of tap weight and *w*_*k*_(*n*) indicates the *k*th filter weight at *n* time index. The final weight updated equation for LMS algorithm [39] is given in vector form as

(10) w(n+1)=w(n)+μ[u(n)e(n)].

Accordingly, for FLMS algorithm, filter weight update equation for *k*th tap weight is written with inclusion of fractional term as

(11) wk(n+1)=wk(n)−μ∂C(n)∂wk−μfr∂frC(n)∂wkfr,

where fr represents the fractional order which is generally taken as real value between 0 and 1, and *μ*_fr_ is fractional step size parameter. The final weight updated equation for *k*th tap in case of FLMS algorithm is written as [33]

(12) wk(n+1)=wk(n)+μe(n)u(n−k)+μfre(n)u(n−k)1Γ(2−fr)wk1−fr(n).

The detailed derivation of (12) can be seen in [33, 40].

### 3.2. Third-Order Volterra Least Mean Square (VLMS) Algorithm

In this section, brief description of third-order Volterra is presented. Volterra model is widely used in many applications of nonlinear systems including system identification, echo cancellation, acoustic noise control, and nonlinear channel equalization and is also used in transmission channels to compensate the nonlinear effects [41–43].

The governing mathematical relations for Volterra series for a causal discrete time nonlinear system having input *u*[*n*] and output *y*[*n*] are introduced by Schetzen, in 1980, and given as [42, 44]

(13) y[n]=∑r=1N∑k1=0M−1…∑kr=0M−1wr[k1,…,kr]u[n−k1]⋯u[n−kr],

where *N* represents the degree of nonlinearity in the model, *M* is the filter memory, *w*_*r*_[*k*_1_,…, *k*_*r*_] is the *r*th-order Volterra kernel. By taking *N* = 3 in (13), the input-output expression for third-order Volterra filter is given as

(14) y[n]=w0+∑k1=0M−1w1[k1]u[n−k1]+∑k1=0M−1∑k2=0M−1w2[k1,k2]u[n−k1]u[n−k1]+∑k1=0M−1∑k2=0M−1∑k3=0M−1w3[k1,k2,k3]       ×u[n−k1]u[n−k2]u[n−k3],

here *w*_3_[*k*_1_, *k*_2_, *k*_3_] is the third-order Volterra kernel of the system. In case of symmetric kernels having memory *M*, then coefficient *M*(*M* + 1)(*M* + 2)/6 is required for third-order kernel [44]. For the third degree of nonlinearity with memory *M*, the volterra kernel coefficient vector **W** is given as:

(15) Wk(3)T=[wk3[0,0,0]wk3[0,0,1]⋯wk3[M−1,M−1,M−1]].

The corresponding input vector **U** for *M* = 3 is written as

(16) U(3)T=[u3[n]u2[n]u[n−1]⋯u[n]u2[n−2]u3 ×[−1]⋯u[n−1]u2[n−2]u3[n]].

The weights update equation for third-order VLMS is given as

(17) Wk+1(3)=Wk(3)+μekUk(3),

where *e*_*k*_ is the error and *μ* is the step size parameter. For the detail description of VLMS, interested readers are referred to [44]. 

### 3.3. Kernel LMS (KLMS) Algorithm

Pokharel et al. have developed the least mean square (LMS) adaptive algorithm in kernel feature space known in the literature as kernel least mean square (KLMS) algorithm [45]. The basic idea of KLMS algorithm is to transform the data from the input space to a high-dimensional feature space. The importance, fundamental theory, the definition of mathematical term, and applications can be seen in [46–49]. 

The KLMS algorithm is a modified version of LMS with introduction of kernel feature space, and its weight updating equation is written as

(18) ω(n+1)=ω(n)+2μe(n)Φ(u(n)),

where *e*(*n*) represents the error term similar to (8) but for KLMS, filter output *y* is computed as

(19) y(n)=〈ω(n),Φ(u(n))〉,

here 〈·, ·〉 represents inner product in the kernel Hilbert space and Φ is a mapping which transforms input vector **u**(*n*) to high-dimensional kernel feature space such that

(20) 〈Φ(u(j)),Φ(u(n))〉=〈κ(·,u(i)),κ(·,u(n))〉=κ(u(j),u(n)),

where Φ(**u**(*n*)) = *κ*(·, **u**(*n*)) defines the Hilbert space associated with the kernel and can be taken as a nonlinear transformation from the input to feature space. Using (20) in (19) gives

(21) y(n)=μ∑j=0n−1e(j)κ(u(j),u(n)).

Equation (21) is called the KLMS algorithm and further detail about the procedure for the derivation of the algorithm is given in [45, 46].

In this study we will only consider most widely used Mercer kernel which is given by translation invariant radial basis (Gaussian) kernel as

(22) κ(u,v)=exp⁡⁡(−||u−v||2σ2).

## 4. Simulations and Results

In this section, results of simulations are presented for two case studies of INCAR model using proposed FLMS, VLMS, and KLMS algorithms. The parameter estimation is carried in both studies by taking different levels of signal-to-noise ratio (SNR) and with various step size **μ** parameters. Moreover, FLMS operates based on different values of fractional orders.

### 4.1. Case Study 1

The INCAR model for this case is taken as follows:

(23) P(z)y(t)=Q(z)u−(t)+v(t),P(z)=1+p1z−1+p2z−2=1+1.35z−1−0.75z−2,Q(z)=q1z−1+q2z−2=z−1+1.68z−2,u−(t)=f(u(t))=a1u(t)+a2u2(t)+a3u3(t)=u(t)+0.50u2(t)+0.20u3(t),θ=[θ1,θ2,θ3,θ4,θ5,θ6,θ7,θ8]T=[p1,p2,a1,a2,a3,q2a1,q2a2,q2a3]T=[1.35,−0.75,1.00,0.50,0.20,1.68,0.84,0.336]T.

In numerical experimentation, the input *u*(*t*) is taken as persistent excitation signal sequence with zero mean and unit variance, and *v*(*t*) is taken as a white noise sequence with zero mean and constant variance. Before applying the design methodology, a figure of merit or fitness function is developed based on estimation error as

(24) ε=||w(n)−θ||||θ||,

where **w**(*n*) is vector of adaptive parameter for INCAR model based on *n*th iteration of the algorithm and vector for the true or desired values is represented by ***θ***. Now the requirement is to find weight vector **w** such that the value of fitness function given in (24) approaches zero, and, consequently, the **w** approaches ***θ***. 

**Figure 1:**
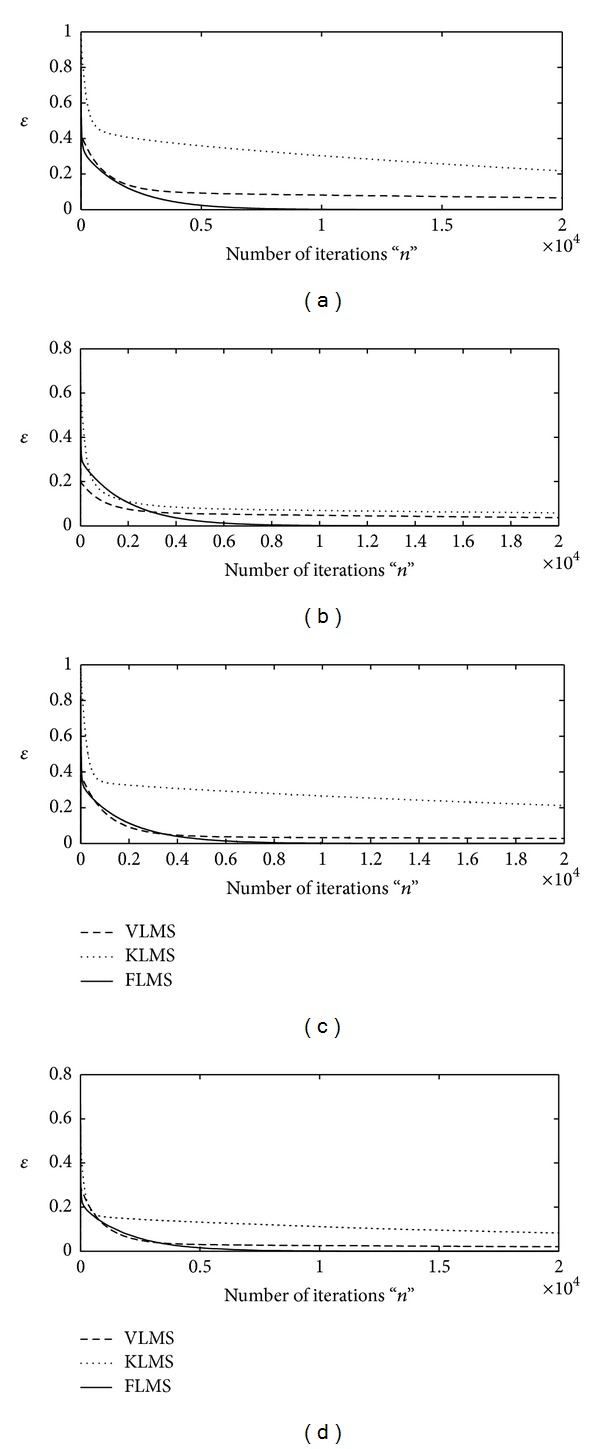
Iterative adaptation of merit function by VLMS, KLMS, and FLMS for fr = 0.5 algorithms for *μ*∈ (10^−04^, 10^−08^); (a) for SNR = 30 dB, (b) for SNR = 20 dB, (c) for SNR = 10, and (d) for SNR = 3 dB.

**Figure 2:**
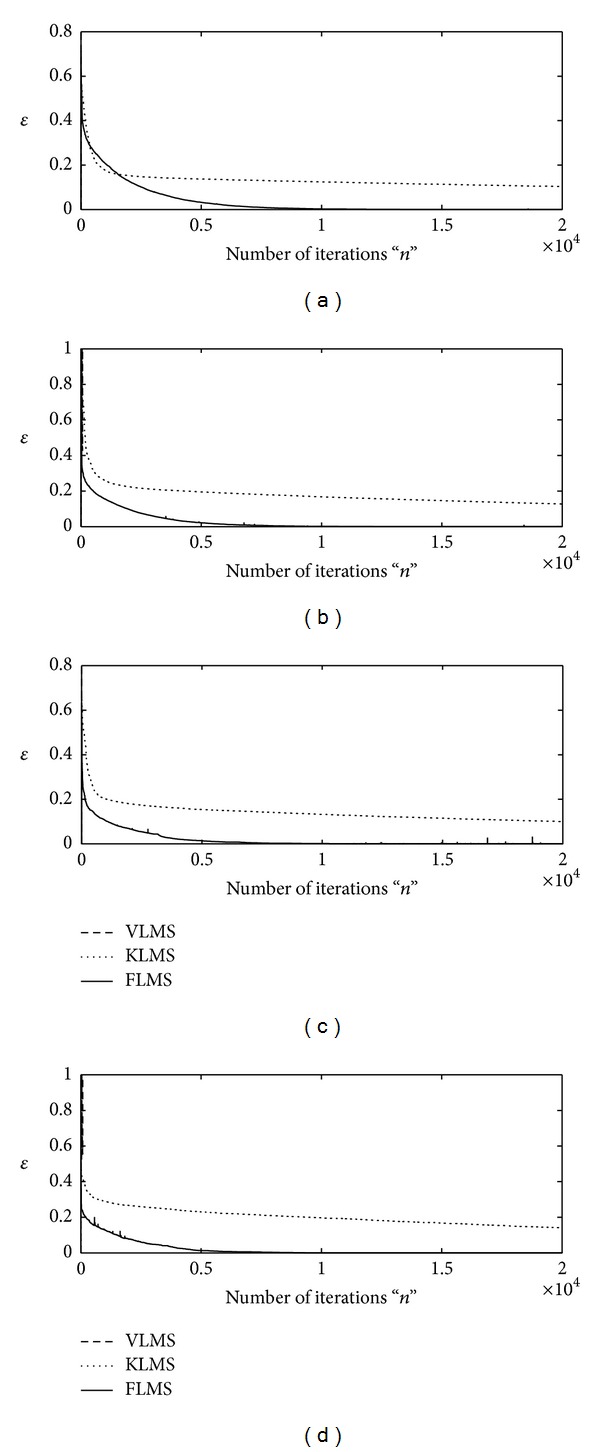
Iterative adaptation of merit function by VLMS, KLMS, and FLMS for fr = 0.5 algorithms for *μ*∈ (10^−03^, 10^−05^); (a) for SNR = 30 dB, (b) for SNR = 20 dB, (c) for SNR = 10, and (d) for SNR = 3 dB.

The proposed adaptive algorithms based on FLMS, VLMS, and KLMS are applied to find the optimal weight vector **w** for INCAR system using sufficient large number of iteration, that is, *n* = 20000. Two types of step size variation strategy are adopted for each algorithm. Firstly, up to *n* = 1000 iterations, the larger values of step size parameter are taken, that is, *μ* = 10^−04^, for fast convergence and for remaining iteration smaller value of step size is used, that is, *μ* = 10^−08^, for the stability. Secondly, initially the step size is taken as *μ* = 10^−03^and later on *μ* = 10^−05^ for *n* > 1000. The design schemes are evaluated for INCAR models based on four different levels of signal-to-noise ratio, that is, 30 dB, 20 dB, 10 dB, and 3 dB. The iterative results of each algorithm against the values of merit function are shown graphically in Figures 1 and 2 for first and second strategy of **μ**, respectively, for all four variants of SNR. It is found that for higher values of SNR and lower values of step size, all the three algorithms are convergent but the accuracy and convergence of the FLMS algorithm are much better than those of VLMS and KLMS. Moreover, with the increase in step size VLMS algorithm diverges, while efficiency of both KLMS and FLMS algorithms increases and remains convergent. 

**Figure 3:**
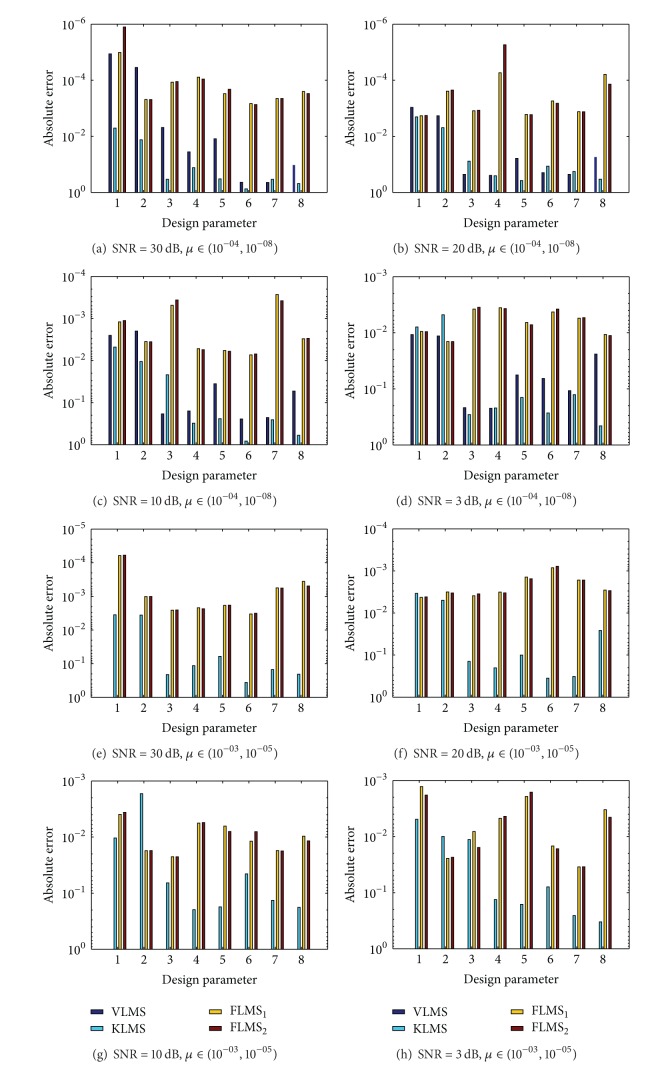
Comparison on the basis of absolute error from true values for INCAR model in case study 1.

The design parameters of INCAR model obtained with adaptation procedure of VLMS, KLMS, and FLMS_1_ for fr = 0.5 and FLMS_2_ for fr = 0.75 are listed in Tables 1, 2, 3, and 4 for SNR = 30 dB, 20 dB, 10 dB, and 3 dB, respectively, for both step size strategies. The values of mean square error (MSE) from true parameters of INCAR model are calculated and its values are also tabulated in Tables 1, 2, 3, and 4 for each algorithm. The values of absolute error (AE) for each element of the design parameter are calculated from reference value of INCAR model and results are presented in Figure 3 for each variant of SNR and for both step size strategies. In order to broaden the small difference in the values, results are plotted on semilog scale. It is seen from the results presented that for high SNR values, like 30 dB, the values of MSE for FLMS_1_ and FLMS_2_ are of the order 10^−07^ to 10^−06^ and for low SNR values like 3 dB the values of MSE are around 10^−04^ to 10^−05^ for FLMS algorithm. Moreover, for increased values of step size, that is, *μ* ∈ (10^−03^, 10^−05^), the VLMS algorithm is not providing the convergent results while both KLMS and FLMS give accurate results. The MSE and AE values of KLMS algorithm are considerably inferior to FLMS algorithm. Generally, it is observed that with decrease in the values of step size parameter, the stability of the algorithm is observed but needs more computational budget to achieve better results.

### 4.2. Case Study 2

Another INCAR system has been taken in this case as

(25) P(z)y(t)=Q(z)u−(t)+v(t),P(z)=1+p1z−1+p2z−2=1+1.35z−1−0.75z−2,Q(z)=q1z−1+q2z−2=z−1+1.68z−2,u−(t)=f(u(t))=a1u(t)+a2u2(t)+a3u3(t)=u(t)+0.50u2(t)−0.20u3(t),θ=[θ1,θ2,θ3,θ4,θ5,θ6,θ7,θ8]T=[p1,p2,a1,a2,a3,q2a1,q2a2,q2a3]T=[1.35,−0.75,1.00,0.50,−0.20,1.68,0.84,−0.336]T.

**Figure 4:**
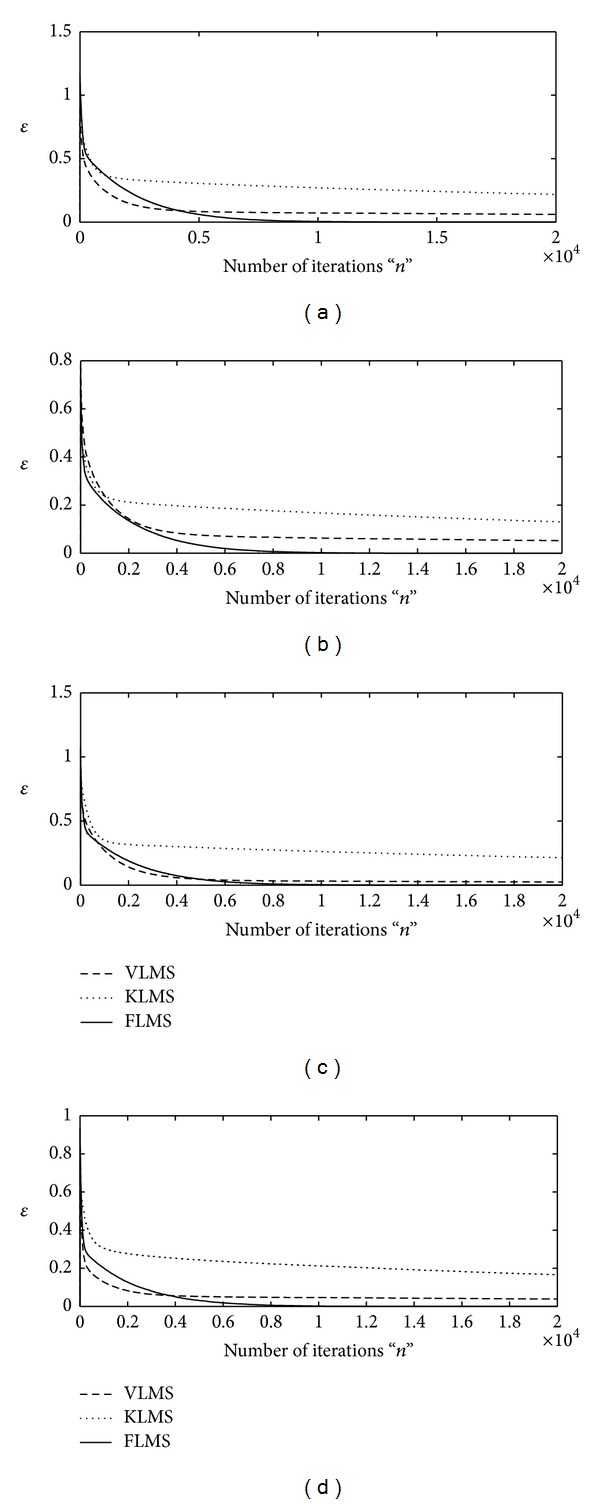
Iterative adaptation of merit function by VLMS, KLMS, and FLMS for fr = 0.5 algorithm for *μ*∈ (10^−04^, 10^−08^); (a) for SNR = 30 dB, (b) for SNR = 20 dB, (c) for SNR = 10, and (d) for SNR = 3 dB.

**Figure 5:**
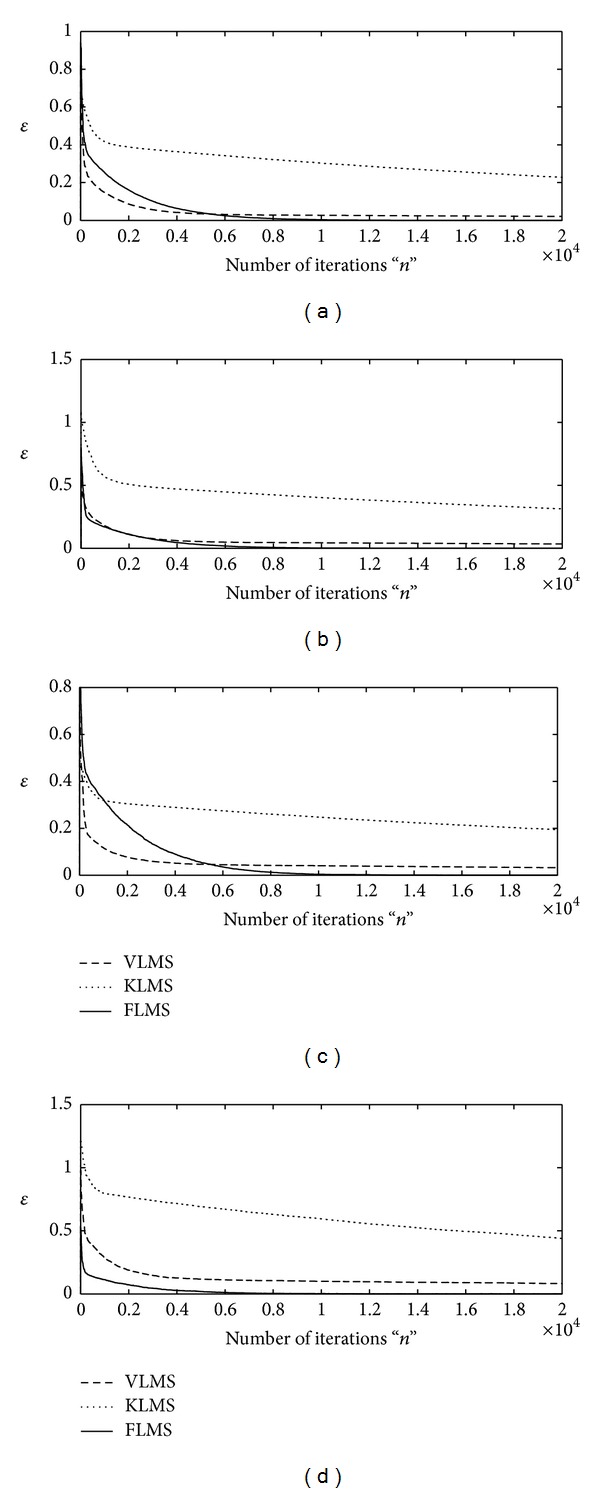
Iterative adaptation of merit function by VLMS, KLMS, and FLMS for fr = 0.5 algorithms for *μ*∈ (10^−03^, 10^−05^) (a) for SNR = 30 dB, (b) for SNR = 20 dB, (c) for SNR = 10, and (d) for SNR = 3 dB.

**Figure 6:**
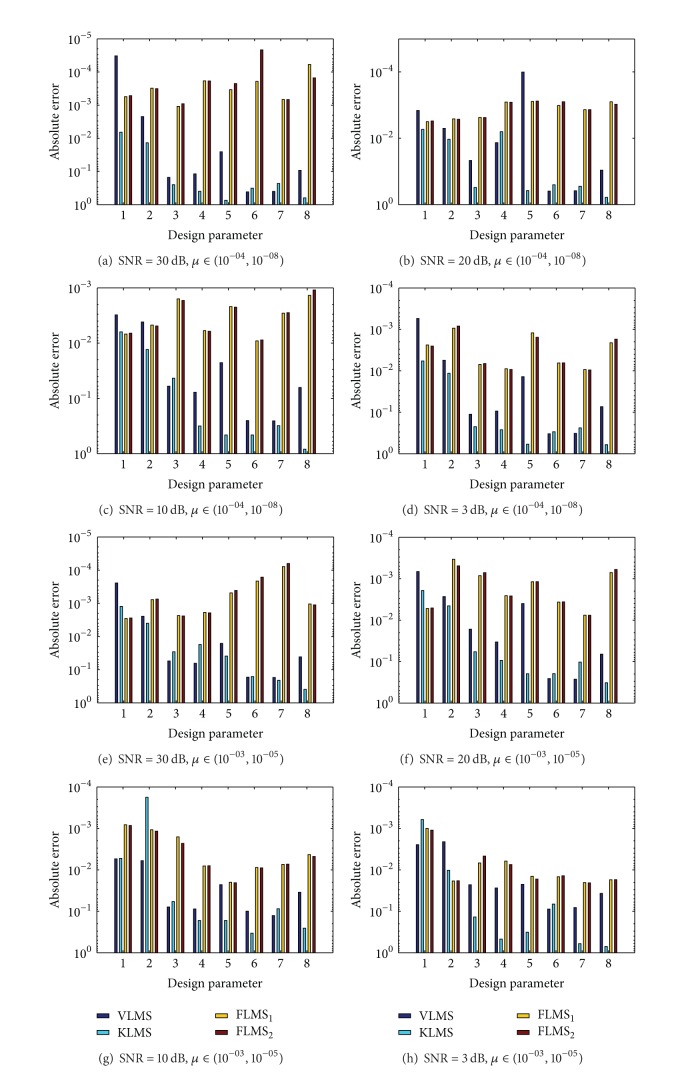
Comparison on the basis of absolute error from true values for INCAR model in case study 2.

The numerical experimentation for this case has been performed on a similar pattern as in the previous case study. The proposed schemes based on FLMS, VLMS, and KLMS methods are applied to find vector of design parameters **w** for INCAR system using sufficient large number of iterations, that is, *n* = 20000. The same types of step size variation strategy and variants of SNR are used for each algorithm in this case as described in the last example. The iterative results of each algorithm against the values of merit function are plotted in Figures 4 and 5 for first and second strategy of **μ**, respectively, for all four variants of SNR. The vector for design parameters of INCAR systems optimized with VLMS, KLMS, and FLMS_1_ for fr = 0.5 and FLMS_2_ for fr = 0.75 are tabulated in Tables 5, 6, 7, and 8 for SNR = 30 dB, 20 dB, 10 dB, and 3 dB, respectively, for both step size strategies. The values of MSE and AE of the proposed schemes from true parameters of INCAR model are calculated and results are given in Tables 5, 6, 7, and 8 and Figure 6, respectively.

It is seen from the results presented that with high SNR values, that is, 30 dB, the values of MSE for FLMS_1_ and FLMS_2_ are around 10^−06^ to 10^−07^ while for low SNR values, that is, 3 dB, the values of MSE are around 10^−04^ to 10^−05^. By increasing the values of step size, that is, *μ*∈ (10^−03^ and 10^−05^), the VLMS algorithm is also giving the convergent results for this case, as well as both KLMS and FLMS provide accurate and convergent results. The MSE and AE values for the KLMS and VLMS algorithms for this case are also found to be inferior from FLMS algorithm. Moreover, it is found that with decrease in the values of step size parameter, the stability in the algorithm is observed but needs relatively more computations to get better results.

## 5. Conclusion

On the basis of the simulation and results presented in the last section, the following conclusions are drawn.

- The adaptive algorithms based on fractional signal processing approach are used effectively for parameter estimation of input nonlinear control autoregressive (INCAR) models for both case studies.
- The variation of step size strategies shows that for smaller and relatively larger value of step size parameter both order of fractional least mean square (FLMS) algorithms provide accurate and convergent results than those of VLMS and KLMS algorithms.
- The variants of signal-to-noise ratio (SNR) in INCAR models show that the performance of all the algorithm decreases as SNR decreases from higher level to lower level, but FLMS algorithm still achieved the values for mean square error around 10^−04^ to 10^−05^ for even SNR = 3 dB.
- Comparative studies between FLMS, VLMS, and KLMS algorithms for each variants of both case studies validate the correctness of the adaptive algorithms based on FLMS algorithm.

In future, one may look for heuristic computing techniques based on genetic algorithms, swarm intelligence, differential evolution, genetic programming, and memetic computing approaches, and so forth, for parameter estimation of INCAR models.

## Figures and Tables

**Table 1:** Comparison of proposed results against true values of INCAR model for 30 dB SNR.

µ	Method	Design parameters								MSE
		*p*_1_	*p*_2_	*a*_1_	*a*_2_	*a*_3_	*q*_2_ *a*_1_	*q*_2_ *a*_2_	*q*_2_ *a*_3_	
(10^−04^, 10^−08^)	VLMS	1.339192	−0.738613	0.783251	0.722700	0.143211	1.614600	0.948003	0.311984	1.46*E* − 02
	KLMS	1.347467	−0.751995	1.185194	0.341222	0.235790	1.435438	1.067026	0.282885	2.19*E* − 02
	FLMS_1_	1.350010	−0.749510	0.999882	0.499921	0.200304	1.679315	0.839550	0.336252	1.36*E* − 07
	FLMS_2_	1.350001	−0.749505	1.000112	0.499909	0.200210	1.679258	0.839549	0.336301	1.44*E* − 07

(10^−03^, 10^−05^)	VLMS	NaN	NaN	NaN	NaN	NaN	NaN	NaN	NaN	NaN
	KLMS	1.353513	−0.746410	1.210424	0.385133	0.139330	1.317242	0.989680	0.541315	3.22*E* − 02
	FLMS_1_	1.349938	−0.748981	0.997433	0.502193	0.201849	1.676682	0.839442	0.335640	3.41*E* − 06
	FLMS_2_	1.349940	−0.748991	0.997454	0.502326	0.201821	1.676806	0.839434	0.335511	3.37*E* − 06

True values		1.350000	−0.750000	1.000000	0.500000	0.200000	1.680000	0.840000	0.33600	

**Table 2:** Comparison of proposed results against true values of INCAR model for 20 dB SNR.

µ	Method	Design parameters								MSE
		*p*_1_	*p*_2_	*a*_1_	*a*_2_	*a*_3_	*q*_2_ *a*_1_	*q*_2_ *a*_2_	*q*_2_ *a*_3_	
(10^−04^, 10^−08^)	VLMS	1.347467	−0.751995	1.185194	0.341222	0.235790	1.435438	1.067026	0.282885	2.19*E* − 02
	KLMS	1.352035	−0.745072	0.923361	0.244504	0.577959	1.563722	0.660224	0.673292	4.67*E* − 02
	FLMS_1_	1.351852	−0.750247	0.998773	0.499946	0.201665	1.679453	0.841320	0.336062	1.23*E* − 06
	FLMS_2_	1.351805	−0.750225	0.998823	0.499995	0.201691	1.679341	0.841338	0.336139	1.22*E* − 06

(10^−03^, 10^−05^)	VLMS	NaN	NaN	NaN	NaN	NaN	NaN	NaN	NaN	NaN
	KLMS	1.353428	−0.745015	1.141632	0.298110	0.300711	1.325873	1.163538	0.362182	3.77*E* − 02
	FLMS_1_	1.345754	−0.753166	0.996107	0.496821	0.201403	1.679157	0.838343	0.338860	8.36*E* − 06
	FLMS_2_	1.345854	−0.753357	0.996484	0.496661	0.201533	1.679222	0.838346	0.338957	8.30*E* − 06

True values		1.350000	−0.750000	1.000000	0.500000	0.200000	1.680000	0.840000	0.33600	0

**Table 3:** Comparison of proposed results against true values of INCAR model for 10 dB SNR.

µ	Method	Design parameters								MSE
		*p*_1_	*p*_2_	*a*_1_	*a*_2_	*a*_3_	*q*_2_ *a*_1_	*q*_2_ *a*_2_	*q*_2_ *a*_3_	
(10^−04^, 10^−08^)	VLMS	1.350924	−0.748145	0.772281	0.747924	0.138729	1.481738	1.066460	0.281078	2.63*E* − 02
	KLMS	1.355082	−0.736640	0.657397	0.631919	0.529419	0.932575	1.184584	0.819516	1.44*E* − 01
	FLMS_1_	1.348795	−0.753541	0.999514	0.505212	0.194240	1.687344	0.839732	0.332946	1.72*E* − 05
	FLMS_2_	1.348876	−0.753596	0.999636	0.505522	0.193962	1.686987	0.839622	0.333021	1.74*E* − 05

(10^−03^, 10^−05^)	VLMS	NaN	NaN	NaN	NaN	NaN	NaN	NaN	NaN	NaN
	KLMS	1.339625	−0.751687	1.065474	0.302732	0.375939	1.634616	0.705183	0.515233	3.88*E* − 02
	FLMS_1_	1.346058	−0.732482	0.977546	0.494359	0.193604	1.691872	0.857421	0.345628	1.80*E* − 04
	FLMS_2_	1.346365	−0.732517	0.977584	0.494478	0.192037	1.688032	0.857678	0.347713	1.79*E* − 04

True values		1.350000	−0.750000	1.000000	0.500000	0.200000	1.680000	0.840000	0.33600	

**Table 4:** Comparison of proposed results against true values of INCAR model for 3 dB SNR.

µ	Method	Design parameters								MSE
		*p*_1_	*p*_2_	*a*_1_	*a*_2_	*a*_3_	*q*_2_ *a*_1_	*q*_2_ *a*_2_	*q*_2_ *a*_3_	
(10^−04^, 10^−08^)	VLMS	1.349988	−0.749965	1.004847	0.536095	0.187753	1.244750	1.281211	0.230035	4.96*E* − 02
	KLMS	1.354804	−0.739470	1.021873	0.808820	−0.040308	0.858857	1.095944	0.934113	1.56*E* − 01
	FLMS_1_	1.340508	−0.735628	1.003795	0.503585	0.206571	1.684274	0.845499	0.346739	6.64*E* − 05
	FLMS_2_	1.340415	−0.735617	1.003511	0.503718	0.207224	1.683801	0.845381	0.347265	6.84*E* − 05

(10^−03^, 10^−05^)	VLMS	NaN	NaN	NaN	NaN	NaN	NaN	NaN	NaN	NaN
	KLMS	1.354922	−0.759970	1.011317	0.632627	0.038110	1.601097	0.584400	0.668535	3.93*E* − 02
	FLMS_1_	1.351284	−0.725432	1.008120	0.504721	0.201932	1.694754	0.805207	0.332677	2.67*E* − 04
	FLMS_2_	1.351821	−0.726599	1.015656	0.504366	0.198380	1.696545	0.805365	0.331465	2.89*E* − 04

True values		1.350000	−0.750000	1.000000	0.500000	0.200000	1.680000	0.840000	0.33600	

**Table 5:** Comparison of proposed results against true values of INCAR model for 30 dB SNR.

µ	Method	Design parameters								MSE
		*p*_1_	*p*_2_	*a*_1_	*a*_2_	*a*_3_	*q*_2_ *a*_1_	*q*_2_ *a*_2_	*q*_2_ *a*_3_	
(10^−04^, 10^−08^)	VLMS	1.353089	−0.745850	0.939847	0.577771	−0.222600	1.428070	1.095959	−0.399576	1.79*E* − 02
	KLMS	1.355430	−0.739217	0.694061	0.493618	0.180732	1.425650	0.558743	0.272328	9.41*E* − 02
	FLMS_1_	1.349448	−0.750309	0.998908	0.499814	−0.199658	1.679808	0.840671	−0.336060	2.79*E* − 07
	FLMS_2_	1.349481	−0.750319	0.999085	0.499812	−0.199776	1.680022	0.840677	−0.336151	2.22*E* − 07

(10^−03^, 10^−05^)	VLMS	1.347530	−0.747896	1.023045	0.527512	−0.177532	1.591528	0.921393	−0.372951	2.20*E* − 03
	KLMS	1.351936	−0.745468	0.941774	0.405908	−0.002987	1.483573	0.737265	−0.011737	2.57*E* − 02
	FLMS_1_	1.347141	−0.749222	1.002343	0.501873	−0.200487	1.679787	0.840078	−0.337043	2.39*E* − 06
	FLMS_2_	1.347230	−0.749253	1.002398	0.501947	−0.200409	1.679837	0.840063	−0.337117	2.40*E* − 06

True values		1.350000	−0.750000	1.000000	0.500000	−0.200000	1.680000	0.840000	−0.336000	

**Table 6:** Comparison of proposed results against true values of INCAR model for 20 dB SNR.

µ	Method	Design parameters								MSE
		*p*_1_	*p*_2_	*a*_1_	*a*_2_	*a*_3_	*q*_2_ *a*_1_	*q*_2_ *a*_2_	*q*_2_ *a*_3_	
(10^−04^, 10^−08^)	VLMS	1.349454	−0.744427	1.111856	0.406555	−0.186142	1.348684	1.163043	−0.409249	3.01*E* − 02
	KLMS	1.355797	−0.738500	0.777289	0.235371	0.385983	1.384078	0.601534	0.270259	1.22*E* − 01
	FLMS_1_	1.353185	−0.747387	0.997633	0.499185	−0.199222	1.681031	0.838613	−0.335203	3.43*E* − 06
	FLMS_2_	1.353038	−0.747304	0.997603	0.499176	−0.199241	1.680798	0.838613	−0.335052	3.37*E* − 06

(10^−03^, 10^−05^)	VLMS	1.344572	−0.743960	0.921542	0.588116	−0.222840	1.580521	0.967188	−0.370809	5.22*E* − 03
	KLMS	1.351239	−0.745972	0.971052	0.517420	−0.160944	1.518107	0.630421	0.060205	2.87*E* − 02
	FLMS_1_	1.355256	−0.749658	1.000849	0.497428	−0.198812	1.683680	0.847624	−0.336714	1.36*E* − 05
	FLMS_2_	1.355106	−0.749508	1.000717	0.497390	−0.198819	1.683638	0.847686	−0.336603	1.35*E* − 05

True values		1.350000	−0.750000	1.000000	0.500000	−0.200000	1.680000	0.840000	−0.336000	

**Table 7:** Comparison of proposed results against true values of INCAR model for 10 dB SNR.

µ	Method	Design parameters								MSE
		*p*_1_	*p*_2_	*a*_1_	*a*_2_	*a*_3_	*q*_2_ *a*_1_	*q*_2_ *a*_2_	*q*_2_ *a*_3_	
(10^−04^, 10^−08^)	VLMS	1.351468	−0.744938	1.047010	0.486303	−0.200102	1.285267	1.225023	−0.427779	3.94*E* − 02
	KLMS	1.356262	−0.736962	0.956832	0.185102	0.259909	1.220949	0.529005	0.495796	1.64*E* − 01
	FLMS_1_	1.356850	−0.745266	1.001581	0.494077	−0.202190	1.670903	0.837135	−0.334630	2.56*E* − 05
	FLMS_2_	1.356608	−0.745109	1.001695	0.493895	−0.202230	1.671239	0.837177	−0.334908	2.48*E* − 05

(10^−03^, 10^−05^)	VLMS	1.349757	−0.747542	0.945167	0.564599	−0.216172	1.511909	1.011739	−0.377157	8.36*E* − 03
	KLMS	1.355306	−0.750178	1.058421	0.333366	−0.033855	1.341960	0.926972	−0.080043	3.08*E* − 02
	FLMS_1_	1.350819	−0.748919	0.998394	0.491951	−0.179965	1.688782	0.847405	−0.340293	7.76*E* − 05
	FLMS_2_	1.350853	−0.748833	0.997694	0.492029	−0.179449	1.688987	0.847312	−0.340771	8.13*E* − 05

True values		1.350000	−0.750000	1.000000	0.500000	−0.200000	1.680000	0.840000	−0.336000	

**Table 8:** Comparison of proposed results against true values of INCAR model for 3 dB SNR.

µ	Method	Design parameters								MSE
		*p*_1_	*p*_2_	*a*_1_	*a*_2_	*a*_3_	*q*_2_ *a*_1_	*q*_2_ *a*_2_	*q*_2_ *a*_3_	
(10^−04^, 10^−08^)	VLMS	1.350033	−0.747776	1.151411	0.382217	−0.174445	1.266046	1.237098	−0.428614	4.69*E* − 02
	KLMS	1.356493	−0.736365	0.748964	0.105174	0.542420	1.362022	0.609956	0.290923	1.65*E* − 01
	FLMS_1_	1.352386	−0.750941	0.992991	0.491026	−0.198780	1.686456	0.849316	−0.338108	3.38*E* − 05
	FLMS_2_	1.352534	−0.750836	0.993316	0.490747	−0.198439	1.686445	0.849539	−0.337726	3.44*E* − 05

(10^−03^, 10^−05^)	VLMS	1.349324	−0.747288	0.983402	0.533690	−0.203984	1.423701	1.105389	−0.402156	1.77*E* − 02
	KLMS	1.350614	−0.760252	1.136970	0.030726	0.119125	1.612852	0.232501	0.372932	1.52*E* − 01
	FLMS_1_	1.351001	−0.731407	0.993154	0.493843	−0.214263	1.694738	0.819746	−0.318643	1.95*E* − 04
	FLMS_2_	1.351107	−0.731604	0.995350	0.492513	−0.216675	1.693931	0.819381	−0.318775	2.01*E* − 04

True values		1.350000	−0.750000	1.000000	0.500000	−0.200000	1.680000	0.840000	−0.336000	
